# Protein complexes in focus

**DOI:** 10.7554/eLife.16156

**Published:** 2016-04-28

**Authors:** Robert M Glaeser

**Affiliations:** Molecular Biophysics and Integrated Bioimaging Division, Lawrence Berkeley National Laboratory, University of California, Berkeley, Berkeley, United Statesrmglaeser@lbl.gov

**Keywords:** cryo-EM, phase plate, single particle analysis, None

## Abstract

A new advance in electron microscopy can reveal highly-detailed structures of protein complexes.

**Related research article** Danev R, Baumeister W. 2016. Cryo-EM single particle analysis with the Volta phase plate. *eLife* **5**:e13046. doi: 10.7554/eLife.13046

**Image** The new phase plate produces images with a higher level of contrast than previously available

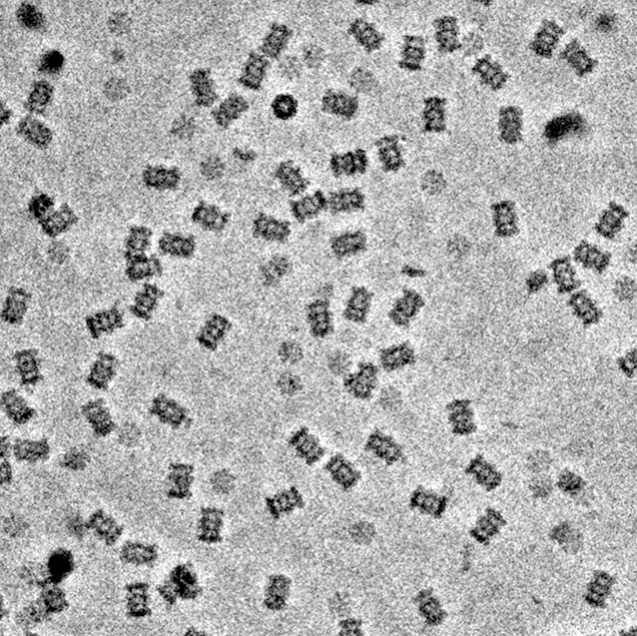

The development of the first electron microscopes in the 1930s made it possible to observe cell structures that could not be seen with traditional light microscopes. However, it has only recently become possible to use electron microscopes to study the structural details of individual protein complexes and other macromolecules (Kühlbrandt, 2014). Improvements in the technique that have made this breakthrough possible – a technique known as single-particle cryo-electron microscopy (cryo-EM; Doerr, 2016) – are a very welcome addition to the other structural tools used in biochemistry and cell biology.

One of the advantages of cryo-EM over other electron microscopy techniques is that samples do not need to be exposed to chemical stains to prepare them for imaging. This means that fragile structures are less likely to undergo modifications before imaging. Unfortunately, if the image of an unstained sample is completely in focus, its features are practically invisible. Microscopists traditionally get around this problem by defocusing the image enough to see the transparent details (Adrian et al., 1984; Taylor and Glaeser, 1976). While out-of-focus images of transparent objects are, paradoxically, far better than in-focus ones, the contrast in such images (that is, the relative difference between the light and dark areas) provides only a ghostly representation of the object being studied. Now, in eLife, Radostin Danev and Wolfgang Baumeister of the Max Planck Institute of Biochemistry show that it is possible to generate highly detailed maps of large protein complexes using images that are in focus (Danev and Baumeister, 2016).

Light microscopy suffered from the same problem until Fritz Zernike discovered that an optical device called a quarter-wave phase plate could be used to produce high-contrast images of all the features in a transparent object, even for in-focus images (Figure 1; Zernike, 1955). While phase plates are easy to produce for light microscopes, making phase plates for electron microscopes has proved to be very difficult. Designs for such phase plates were proposed as early as 1947 (Boersch, 1947), but progress has been slow. 15 years ago, for example, Danev and Kuniaki Nagayama made a phase plate by using a focused-ion beam to drill a tiny hole in a thin carbon film (Danev and Nagayama, 2001; Danev and Nagayama, 2008). However, prolonged use led to a phenomenon known as “charging”, which introduced unwanted phase shifts into the electron beams.

**Figure 1.**
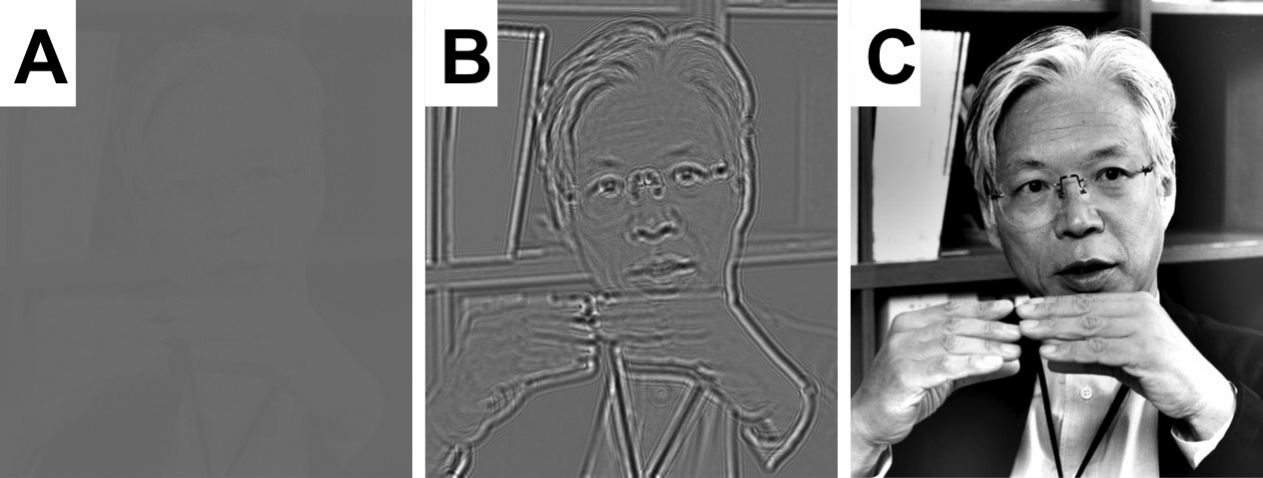
Simulations showing how a quarter-wave phase plate improves the contrast of in-focus images. In these simulations the intensity of the beam (which can be made of light or electrons) remains the same everywhere it passes though the “transparent" object; however, the phase changes significantly where the beam passes through different parts of the object. (**A**) A perfectly focused, bright-field image has almost no contrast because it can only show variations in the transmitted intensity. (**B**) A defocused image is able to capture at least a small part of the changes in the phase, so the contrast is better and the object can be seen. (**C**) Zernike realized that changing the phase of the unscattered portion of the transmitted wave (that is, the part that was not deflected when the wave passed through the object) by 90 degrees greatly improved the quality of the image. The scientist in the photograph is Professor Kuniaki Nagayama, who has played a seminal role in the development of phase plates for use in the transmission electron microscope. Image credit: Dr. Radostin Danev.

A major breakthrough finally happened when Danev and colleagues discovered that charging could itself be exploited to generate the 90 degree phase shift needed in a quarter-wave phase plate (Danev et al., 2014). This new 'Volta' phase plate is already rapidly transforming the field of electron cryo-tomography (Danev et al., 2014; Mahamid et al., 2016).

Now, Danev and Baumeister go on to explore the potential uses of the Volta phase plate in single-particle cryo-EM. The first results are extremely exciting: a three-dimensional density map of a bacterial 20 S proteasome at a resolution of 3.2 Å! This protein complex, which plays a crucial role in degrading old and unwanted proteins, has been previously studied using a variety of structural biology techniques. The fact that the new results agree well with these previous findings is an important first step in testing the new phase plate.

I am personally optimistic that this technology will soon lead to equally good results for protein complexes that have proven to be difficult to study with existing techniques. Similarly, the increased signal-to-noise ratio that the phase plate provides ought to make it possible to obtain highly-detailed structures from many more proteins than is possible now, including proteins as small as, or even smaller than, hemoglobin.

However, Danev and Baumeister identify a challenge that will need to be addressed. To reconstruct the structure of a protein complex, many cryo-EM images need to be merged together using image processing software. Correcting for each image being out-of-focus by a (slightly) different amount is currently a standard part of image processing in cryo-EM, but this is much trickier to do when images are taken as close as possible to being in focus. Although Danev and Baumeister took steps to collect each image as carefully as possible, I worry that some remaining uncertainty might limit the level of detail achieved when the images are processed.

One way to determine the focus more accurately would be to prepare samples on a thin, amorphous-carbon support film. One could then estimate the focus from looking at the support film in the images, although the support film would also increase the amount of noise in the images. This is a step that many researchers who use cryo-EM are unwilling to take. I thus imagine a door of opportunity will open to anyone who comes up with a better alternative.
